# Can the activities of the large scale cortical network be expressed by neural energy? A brief review

**DOI:** 10.1007/s11571-015-9354-0

**Published:** 2015-09-03

**Authors:** Rubin Wang, Yating Zhu

**Affiliations:** Institute for Cognitive Neurodynamics, East China University of Science and Technology, Shanghai, 200237 China

**Keywords:** Neuronal energy, Biophysical energy, Feeding the brain, Energy of the large scale cortical network

## Abstract

This paper mainly discusses and summarize that the changes of biological energy in the brain can be expressed by the biophysical energy we constructed. Different from the electrochemical energy, the biophysical energy proposed in the paper not only can be used to simulate the activity of neurons but also be used to simulate the neural activity of large scale cortical networks, so that the scientific nature of the neural energy coding was discussed.

An important research project in brain science is how to encode and decode external information in the brain. This issue, confusing our understanding of perception, has long been a concerned of cognitive neuroscientists, computing neuroscientists and artificial intelligence scientists. Perceptual neural coding has an enormous impact on the comprehension of working mechanisms in the brain and the application prospects of artificial intelligence.

Neural coding and decoding are the most important and yet challenging research subjects in neuroscience (Amari and Hiroyuki 2005; Danielle et al. 2009; Sengupta et al. 2010; Feldman 2009; Wagatsuma and Yamaguchi 2007). Today, there is not well-established theory of neural coding and decoding to support the investigation of global behaviors of brain activities, and many problems in neural coding and decoding are difficult to solve technically (David and Laughlin 2009; Laughlin and Sejnowski 2003; Singer 2009; Fox and Raichle 2007; Hipp et al. 2011; Feldman 2013).

We can prove that the neural activities and operations in the brain are subordinated to the principle of depleting minimized energy while maximizing the efficiency of signal transmission (Zheng and Wang 2012; Wang et al. 2015a). In other words, the activities of the nervous system are confined to energy minimization, with either sup-threshold or sub-threshold stimulus, which reflects the economic nature of the nervous system. In a neural network, the efficiency of signal transmission is achieved by maximizing the utilization of energy, resulting in the high efficiency of the nervous system.

However, it is difficult to describe the quantitative relationship between the biological energy and the activities patterns of biological energy in the brain (Wang and Wang 2014, 2015; Attwell and Laughlin 2001).

Up until now, research on energy consumption in the nervous system has been limited to experiments due to the brain complexity (Raichle 2010). There are also some discussion on the quantitative calculation and analysis of the energy of the neuron, but these discussions belong to the electrochemical energy (Moujahid et al. 2011, 2014; Alle et al. 2009). At the level, neuronal energy cannot be coupled with energy coding at networks level, so, calculation of the electrochemical energy cannot be used in the study of nervous energy coding, but it is helpful to understand the mechanism of biological energy consumption in the brain.

In order to be able to use the quantitative method to describe the biological energy and the relationship between changes of biological energy and neural information processing in cortex, we constructed a biophysical energy to simulate the biological energy in the brain. And through changes of the biophysical energy to describe the relationship between biological energy in the brain and neural information processing in cerebral cortex, it follows that the various firing patterns of energy are quantitatively given in the evolution procedure of neural energy. Due to the unique corresponding relationship between the various distribution patterns of neural energy and the oscillation mode of the network, we have proposed the new concept of neural energy encoding in recent years (Wang and Zhang 2006, 2007; Wang et al. 2008, 2009).

To sum up, there are limitations in single neuron coding and population coding, so there is a great need for an effective theory that describes neural coding in the brain, cognitive coding, and behavioral coding at various levels to help guide neuroscience experiments. The neural activities and operations in the brain follow the principal of minimization of energy and maximization of transmission efficiency (Wang and Zhang 2007). Specifically, the activity of neural systems with sup-threshold or sub-threshold stimuli adheres to the minimization of energy, reflecting the economics of the neural system. The transmission efficiency of signals in neural networks is obtained by maximizing energy efficiency, which is illustrated in the high efficiency of neural systems. In addition, Japanese scientists initially proposed that information can be converted into energy, which they tested with a physical experiment. In the lab, they made a nano-ball climb the “ladder” created by an electric field. It should be noted that the energy needed during the process is converted from the information about the direction of the movement at any given time (Tayabe et al. 2010). In other words, the ability of the ball to climb the ladder is determined by the information of “its position,” without the researchers applying any external force (such as injecting new energy, etc.). This means that the transformation from information to energy is achieved in the experiment, that is to say, information can be expressed by energy (Wang and Wang 2014; Wang and Zhang 2006; Wang et al. 2008). This scientific idea provided a theoretical basis for neural energy coding. Based on the operation of the brain and the scientific notion that information can be converted into energy, Wang proposed the hypothesis of neural energy coding, which attempts to explain the regular pattern in the process of perceptual cognition and demonstrate the neural dynamic mechanism of energy evolution for neural activity in the cortex (Wang and Wang 2014, 2015; Wang and Zhang 2006; Laughlin and Sejnowski 2003). The reason why the amplitude of blood oxygen level (BOLD)dependent contrast signal of functional magnetic resonance imaging (fMRI) is studied relatively little is that the relationship between a subject’s performance and signal amplitude changes with the familiarity of the task. For example, in the case of a complex cognitive task, high-amplitude signals are initially associated with good performance, but after subjects are familiar with the task, good performance may not lead to high-amplitude signals. Therefore, although neural energy coding is scalar, it can reflect the regular pattern of brain activity based on the principals of brain operation (Wang and Zhang 2007). In the study of neural energy, Wang and Zhang demonstrated that neural action potentials and corresponding energy changes with computation of neural energy under sup-threshold or sub-threshold stimuli (Wang and Wang 2014; Wang and Zhang 2006; Wang et al. 2009). An important finding was that the neuron absorbs energy first and then consumes energy during the firing action, which modifies the traditional idea that the neuron completely consumes energy. The finding also explains a neurophysiological phenomenon, not discussed until now, that cerebral blood flow significantly rises with a small increase in oxygen consumption when the neuron is activated. Based on the above-mentioned studies, Wang discussed the following issues: (a) the membrane potential energy function of single neural activities under a variety of coupling; (b) total energy function of neural populations; (c) energy flow in a network consisting of neural populations; and (d) neural energy coding based on energy changes with respect to time (Wang and Wang 2014, 2015; Wang and Zhang 2006; Wang et al. 2008). Wang et al.’s studies showed that the energy method has some advantages in studying neural coding and related neural activities, which includes five aspects. First, based on neural networks, the important properties of time-dependent neural energy coding is the unique correspondence between the neural membrane potential and neural energy, which means that neural information coding can be expressed by neural energy coding. Second, neural networks are self-organizing, so that they are divided into several neural groups corresponding to the regions of energy costs in the synchronized neural network when stimulation starts. In other words, neural energy encoding can automatically assign the neural energy in accordance with each frequency of the synchronized oscillation corresponding to the regional ratio in the total energy of the network, and separate each neuronal group from the network. Notably, it is very important to analyze the functional neural network model, especially to study the perceptual cognitive and the advanced cognitive functions. Third, the neural energy can be superimposed, which promotes modeling and computational analysis for a higher-dimensional complex nonlinear neural network that consists of a large number of neurons. It is important that the total superimposed energy of the neural network can reflect the state of the synchronized oscillation in the global network at the same time. Fourth, neural energy can be a carrier linking neural information to cerebral blood flow. The periodical alternation of positive and negative energy in the neural network can indicate that the energy supply in the cerebral blood flow and the energy consumed in the neural population, proving that there must be a coupling relationship between neural information processing and the change of blood flow, which provides a theoretical basis for neural modeling in the future. Fifth, neural energy coding probably reduces the cost of analysis greatly. Wang and Zhang explored why neural energy is an effective instrument for probing the global activity of brain, and they indicated that neural coding that carries information is related to energy efficiency. Just as the plasticity of nervous system potentially leads rare resources to a needy region, neural energy can lead to energy consumption in a region where the neural information processing is most required. This viewpoint explains the reason why neural energy is an effective method for exploring the global activity of the brain. In recent studies, Wang et al. conducted a preliminary study of the mechanism of cognitive neural dynamics for synchronized oscillation with respect to energy. The cognitive and conscious activity of the brain actually controls the cerebral operations by means of the transformation between functional networks and cognitive networks. Therefore, a systematic method is needed to study how to build relationships between neural computations in local networks and overall neural activity modes. Synchronized oscillations led by dynamic balance and functional combinations of inter-regional networks are originated from the rhythmic movements of the neural population with a different frequency. Neural energy coding can simulate this type of neural activity, so that the relationship between local networks and overall neural activity in nervous system can be studied in terms of energy (Wang and Wang 2014; Alle et al. 2009).

Moreover, Wang and Zheng also have studied the physiological mechanism of the phenomenon of decreased neural consumption and negative energy in the computation, indicating that it is contra fluxion caused by neural activities that lead to the above phenomenon (Laughlin and Sejnowski 2003). Based on the anatomical structure of an astrocyte, this type of cell is suitable to regulate blood flow in accordance with the neural activities in the region (Huettel et al. 2009; Wang et al. 2015b; Zheng et al. 2014; Parri and Crunelli 2003). According to this theory, the increase of calcium in an astrocyte leads to angiectasis, and then the blood flow increases oxyhemoglobin, from which neurons acquire energy, which flows into tissue (Sokoloff 2008). However, at this moment, the oxygen consumption of neurons does not rise proportionately with the increase in blood flow and the oxygen it contains (Peppiatt and Attwell 2004). By means of positron emission tomography(PET), Fox et al. observed that the Oxygen Extraction Fraction (OEF) decreases from 40 % at the resting state to 20 % when an event is triggered, which means that 80 % of oxygen is not physiologically metabolized during the processing of the event. This indicates that the energy consumed during neural activation is trivial compared with the resting state, and that the congestion reaction of cerebral blood flow is affected by the non-oxidation of metabolites (lactic acid for example), while masses of oxygen and biological energy are consumed during the conduct of action potential. Consequently, oxyhemoglobin changes into deoxyhemoglobin during this process. Notably, the model of neural energy coding proposed by Wang reflects a physiological mechanism of neural activity, expressing the basic rule of cerebral activities during the process of perceptual cognition (Zheng et al. 2014). Large amounts of energy originating from oxygen and glucose in the blood are needed to maintain brain operations, so that the brain’s ability to process information is limited by the disposable energy. For nearly a century, people generally believed that blood flow increases when the neurons in a region are activated, thereby reflecting the level of brain activity, which is the theoretical basis for nuclear magnetic resonance (NMR) (Peppiatt and Attwell 2004). The relationship between the functional stimulation and regulation of blood flow is unclear. However, local functional stimulation and local energy metabolism take place in the same cell, so that local energy metabolism is more closely related to local functional stimulation compared with blood flow (Moore and Cao 2008). Neural energy computation can provide reasonable explanations for this unknown problem, and meanwhile, lay a solid foundation for the study of coupling models relating neural firing and blood flow.

Since the neural structure of the brain is complex and has multi-level, the emergence of the cognitive function is determined by the multi-level coupling and coordination of various brain regions. To reveal the neural coding in the cortex, we need to study it from a different perspective, at a different level, as well as in combination of the different levels. Especially, an effective theory of neural coding is proposed for the global brain activities.

In a series of papers on neural energy we have published (Wang and Wang 2014, 2015; Wang and Zhang 2006, 2007; Wang et al. 2008, 2009), neural information can be expressed by energy which means that (1) Neural information can be expressed by the energy based on the level of molecules, neurons, networks, cognition and behavior as well as on the combination among these levels, that is to say, the energy can be used to unify the neural models from various levels.(2) Neural information can be analyzed according to the neural energy combined with the distribution mode of membrane potentials. (3) The mode of network coupled oscillation can be varied, while the coupled oscillation of the neural network has a unique corresponding relationship with the network energy oscillation. While the large scale neural network modeling and numerical analysis cannot be obtained due to the complexity of high dimensional nonlinear coupled mode, neural energy coding can be used to study neural processing which simplifies the study on neuroinformatics.

The major characteristic of neural energy coding based on network is that there is a unique corresponding relationship between neuron membrane potential and neural energy. Therefore, the energy flow varied with time can reflect the conduction of information flow in network. In other words, neural information coding can be expressed with neural energy coding. It is necessary to explain that to express neural information with neural energy is still a conjecture. It can be confirmed that, however, the energy variation patterns constraining all of neural activities will also vary with varied membrane potential or oscillation patterns of network correspondingly. Our recent research suggests that mental exploration can also be expressed by neural energy. In the new work, based on the concept of mental exploration, neural energy coding theory has been applied to the calculation model so as to solve the path search problem: energy field is constructed in the model on the basis of the firing power of place cell clusters, and energy field gradient is calculated which can be used to study mental exploration problem. The study shows that the new mental exploration model can efficiently find the optimal path, and present the learning process with biophysical meaning as well. This idea verifies the efficiency of energy coding, which also provides the theoretical basis for the neural dynamics mechanism of spatial memory. In conclusion, it is absolutely possible to express neural information processing with neural energy. It is necessary to be stressed that large-scale neural network model can be established with neural energy coding in macroscopic (in all layers of molecule, neuron, network, and behavior, and combination of all layers), so this model can neglect the synaptic coupling between neurons, simplifying the non-linear and complex network model with high-dimensional coupling into a simpler model. The method in this study aims at converting a complex problem that is difficult to be mathematically solved into a problem that can be solved by calculation and stimulation. Meanwhile, the energy method follows principles of minimum energy and maximum efficient of neural information transmission in coding. Therefore, this method provides a brand new angle to study neural information processing of brain in overall view.
